# TGF-β1 enhanced myocardial differentiation through inhibition of the Wnt/β-catenin pathway with rat BMSCs

**DOI:** 10.22038/ijbms.2020.42396.10019

**Published:** 2020-08

**Authors:** Yang Lv, Xiu-juan Li, Hai-Ping Wang, Bo Liu, Wei Chen, Lei Zhang

**Affiliations:** 1 Department of Histology and Embryology, Hebei North University, Zhangjiakou, Hebei, China; 2 Department of Histology and Embryology, Hebei Medical University, Shijiazhuang, Hebei, China; 3 Department of Pathology, the First Affiliated Hospital of Hebei North University, Zhangjiakou, Hebei, China

**Keywords:** Bone marrow mesenchymal - stem cells, Cardiomyocytes, Cell differentiation, Transduction, Transforming growth factor - beta1, Wnt signaling pathway

## Abstract

**Objective(s):**

To investigate and test the hypotheses that TGF-β1 enhanced myocardial differentiation through Wnt/β-catenin pathway with rat bone marrow mesenchymal stem cells (BMSCs).

**Materials and Methods:**

Lentiviral vectors carrying the TGF-β1 gene were transduced into rat BMSCs firstly. Then several kinds of experimental methods were used to elucidate the related mechanisms by which TGF-β1 adjusts myocardial differentiation in rat BMSCs.

**Results:**

Immunocytochemistry revealed that cTnI and Cx43 expressed positively in the cells that were transduced with TGF-β1. The results of Western blot (WB) test showed that the levels of intranuclear β-catenin and total β-catenin were all significantly decreased. However, the cytoplasmic β-catenin level was largely unchanged. Moreover, the levels of GSK-3β were largely unchanged in BMSCs, whereas phosphorylated GSK-3β was significantly decreased in BMSCs. When given the activator of Wnt/β-catenin pathway (lithium chloride, LiCl) to BMSCs transducted with TGF-β1, β-catenin was increased, while phosphorylated β-catenin was decreased. In addition, cyclinD1, MMP-7, and c-Myc protein in BMSCs transducted with Lenti-TGF-β1-GFP were significantly lower.

**Conclusion:**

These results indicate that TGF-β1 promotes BMSCs cardiomyogenic differentiation by promoting the phosphorylation of β-catenin and inhibiting cyclinD1, MMP-7, and c-Myc expression in Wnt/β-catenin signaling pathway.

## Introduction

As a serious disease which leads to permanent loss of cardiomyocytes, acute myocardial infarction (MI) has become one of the main causes of morbidity and mortality worldwide (1). Patients with MI often have poor prognosis, which urgently needs new therapies to repair myocardium with non-function (2). In recent years, scientists put great energy and effort into repairing damaged myocardium by stem cells (3).

Given their capacity for expansion, multipotency and low immunogenicity, bone mesenchymal stem cells (BMSCs) have become some of the highest potential cells for tissue reparations over the last decade (4, 5). BMSCs are able to differentiate into adipocytes, chondroblasts, or osteocyte under different stimulation conditions. Moreover, BMSCs can also differentiate into other types of cells, such as neurons and cardiomyocytes (CMCs) *in vivo *and *in vitro* (6, 7). Through random demethylation, 5-azacytidine becomes a typical inducer that enhances differentiation between BMSCs into CMCs both* in vitro* and *in vivo* (8, 9). However, it is toxic and the differentiation ratio is low (10). Furthermore, the exact molecular mechanisms of differentiation of BMSCs into CMCs are still not understood.

As a multifunctional cytokine, transforming growth factor beta 1 (TGF-β1) can be involved in cell growth, differentiation, and survival (11). Wnt/β-catenin signaling can also be involved in cell growth, development, and metabolism (12). It can control cardiac progenitor cell renewal and differentiation (13). With transcriptional factors of T-cell factor/lymphoid enhancer factor (TCF/LEF) family, β-catenin forms a complex in the nucleus, then activates target gene transcription. Studies show that both Wnt/β-catenin and TGF-β1 signaling stimulate and cooperate with each other, which plays an important role in controlling cell development (14).  Si *et al.* (15) investigated the role of PlncRNA-1 in hair follicle stem cells (HFSc). There results showed that PlncRNA-1 may promote HFSc proliferation, differentiation by up-regulation of TGF-β1-mediated Wnt/β-catenin signaling. Xiang *et al.* (16) believed that through activating Wnt/β-catenin signaling, oxidored nitro domain containing protein 1 can activate human hepatic stellate cells and contribute to liver fibrosis *in vitro*. In this study, we hypothesized that Wnt/β-catenin signaling involved in the process of TGF-β1 enhances myocardial differentiation with rat BMSCs. To test our hypothesis, the TGF-β1 gene was transducted into rat BMSCs firstly. Then we used confocal and electron microscopy, WB, immunocytochemistry, and real-time quantitative polymerase chain reaction (RT-qPCR) to discuss whether TGF-β1 regulates Wnt/β-catenin signaling and enhances myocardial differentiation in rat BMSCs.

## Materials and Methods

***Animals***

Fifteen Sprague-Dawley (SD) rats (3-week-old, 35–45 g) were used for cell isolation experiment. The experiments were performed according to the protocols by the Institutional Animal Care and Use Committee of Hebei North University (2017-1-9-13).

***Isolation and culture of BMSCs***

As described in our previous study (17), bone marrow that was separated from the SD rats’ limb bones was collected and diluted with 5 ml IMDM-LG (Gibco BRL, USA) complete medium. The medium was replaced every 3 days. When the cell density reached more than 90%, they were digested and transferred to fresh flasks at a ratio of 1:2.

***Flow cytometry (FCM) analysis for surface marker on cells in vitro***

FCM analysis was performed as previously described (18). Fourth generation BMSCs were identified by FCM (FACSAriaTM II SORP; BD Biosciences, USA). BMSCs were harvested and suspended in culture medium (IMDM-LG medium containing 15% FBS) at a concentration of 1x10^6^ cells/ml. Fluorescein isothiocyabate-or PE-conjugated CD29 (1:10; cat. no. 562154), CD90 (1:10; cat. no. 561404) and CD45 (1:20; cat. no. 561867) were used to identify the cultured cells. FCM data were analyzed using FlowJo software (Tree Star INC., USA). 

***Multi-directional differentiation of BMSCs***

The multilineage differentiation potential of BMSCs was detected using adipogenic, osteoblast, and chondrogenic differentiation *in vitro* (19, 20). To induce osteoblasts, BMSCs were subjected to a standard osteogenic medium (OM) composed of 1×10^-8^ mol/l dexamethasone (Sigma-Aldrich, USA), 5×10^-2^ g/lascorbic acid (Sigma-Aldrich, USA), and 1×10^-2^ mol/l β-glycerol phosphate (Sigma-Aldrich, USA) for 21 days. Then, to evaluate calcium accumulation, cells were incubated at RT for 30 min with Alizarin Red S (pH 4.1, Sigma-Aldrich, USA).

For adipogenesis, cells were treated in IMDM-LG (Gibco BRL, USA) complete medium, with 10 mg/l insulin, 1 μM dexamethasone, 200 μM indomethacin (Sigma-Aldrich, USA), and 500 μM 3-isobutyl-1-methylxanthine (IBMX, USA) for 14 days. At last, cells were analyzed by Oil Red O staining (Sigma-Aldrich, USA).

For chondrogenesis, cells were cultured for 21 days in IMDM-LG complete medium, with 50 mg/l ascorbic acid (Sigma-Aldrich, USA), 10 μg/l TGF-β3 (Peprotech, Rocky Hill, NJ, USA), 10 mmol/l dexamethasone (Sigma-Aldrich, USA), and 100 μg/l IGF-1 (PeproTech). At last, cells were analyzed by Alcian Blue (J&K Scientific, China) staining.

***Exogenous gene transduction of TGF-β1 to enhance myocardial differentiation of BMSCs***

Experimental grouping: cells in each group underwent transduction as follows: 

Group A, BMSCs were transducted with lentivirus carrying green fluorescent protein (GFP)/TGF-β1 (Lenti-TGF-β1-GFP). 

Group B, BMSCs were transducted with an empty vector (Lenti-empty vector-GFP). 

Group C, blank culture group of BMSCs. 

According to the above grouping, lentivirus was transducted into cells (21). Briefly, the 2nd-generation BMSCs were seeded at 1 × 10^5 ^cells/cm^2 ^ in 6-well culture plates and sequentially infected with lentivirus (Odobio (Shanghai) Co., Ltd.) in FBS-free IMDM medium (MOI=100, 150 and 200) using Polybrene (5 μg/ml culture medium; Odobio (Shanghai) Co., Ltd.) for 16 hr. The following day, cells were added with fresh media without virus. At 96 hr after transduction, the 3rd-generation BMSCs were prepared and fluorescence analysis was performed. The efficiency of transduction was estimated by detecting the proportion of GFP positive BMSCs under a fluorescence microscope. 

***Detection of TGF-β1 protein expression in each group using Western blot (WB)***

At 96 hr following transduction, the cellular total protein in each group was collected. Western blot analysis (22) was used to detect TGF-β1 protein expression in each group. The relative expression of protein was calculated by the ratios of the absorbance of target protein (TGF-β1, 1:500, sc-146; Santa cruz, USA) to β-actin (1:1,000, #4967; Cell Signaling Technology, Inc.).

***Analysis of BMSCs-cardiac differentiation by immunocytochemistry***

As described in the previous study (23), BMSCs (1×10^6^) in each group were seeded in 24-well plates containing poly-D-lysine-coated cover-glass. Seventy-two hours later, they were washed with PBS and fixed by 4% paraformaldehyde. The expression of cTnI (1:100; ab-47003; Abcam, Shanghai, China) and Cx43 (1:100; #C0158; anbobio, Shanghai, China) was analyzed using the Streptavidin/peroxidase (SP) immunocytochemistry method. The data was analyzed by Image-pro Plus 6 software (Media Cybernetics, USA). 

***Detection of BMSCs-cardiac differentiation by transmission electron microscopy (TEM)***

TEM detection was performed as previously described (18). Two weeks following transduction, the cells were harvested and fixed by 3% glutaraldehyde and 1% osmium tetroxide. After embedding, the sample was cut horizontally by Ultra-thin sections and stained by uranyl acetate together with lead citrate. Then the ultrastructure was observed using a TEM (JEM-2000EX, Japan).

***Detection of the relationship between myocardial differentiation of BMSC transducted with TGF-***β1*** and Wnt/β-catenin signaling using Western blot***

The first antibody in Western blot used was as follows: β-catenin (sc-7963), GSK3β (sc-81462), p-GSK3β (sc-373800)(all diluted at 1:500; Santa cruz, USA), c-Myc (bs-4963R), cyclinD1 (bs-0623R), MMP-7 (bs-0423R)(all diluted at 1:500; Bioss Biotechnology Co., Ltd, China), and β-actin (1:1,000; #4967; CST, USA).

Fourteen days following transduction, the cellular total protein, nucleoprotein and cytoplasm protein in each group was collected (24). Operations were strictly carried out according to kit instructions (min^TM ^Protein Extraction Kit, SD001/SN002; min^TM ^Cytoplasmic and Nuclear Extraction Kit, SC003, Invent, China). Then Western blot (24) was used to detect GSK3β, p-GSK3β, β-catenin, MMP-7, cyclinD1, and c-Myc protein in each group, which were performed as described previously. The relative expression was calculated by the ratios of target protein absorbance to β-actin (Lamin B1 for nucleoprotein) absorbance.

***Analysis of the relationship between Wnt/β-catenin signaling pathway and myocardial differentiation of BMSC transducted with TGF-β1 using RT-qPCR***

As described in the previous study (23), the transcription factors including MMP-7, cyclinD1, and c-Myc were detected by RT-*q*PCR on day 14 and the changes were normalized to GAPDH levels. Primers 6.0 software was used to design the primers. Primers were listed in Table 1. Cycle threshold (Ct) of each gene was analyzed. The fold-change in each group for target genes was calculated as average value of 2^-∆∆Ct^ ± standard deviation. 

***Analysis of the effect of lithium chloride (LiCl) on the expression of ***β-catenin*** and p-***β-catenin in different ***groups using Western blot***

This experiment also comprised four groups as follows: blank culture group of BMSCs as the control group; single transduction group, BMSCs were transducted with Lenti-TGF-β1-GFP (MOI=150); the LiCl group, BMSCs were treated with 20 mM LiCl; transduction + LiCl group, BMSCs were treated both with Lenti-TGF-β1-GFP (MOI=150) and LiCl (20 mM).

Operations were strictly carried out according to kit (SD-001/SN-002, invent, China) instructions (24), which were performed as described previously. The primary antibodies, β-catenin (sc-7963; Santa cruz, USA) and p-β-catenin (sc-16743-R; Santa cruz, USA), were all diluted at 1:500.

***Statistical analysis***

SPSS 17.0 software (USA) was used for statistical analysis. To compare the difference between groups, one way analysis of variance and *post hoc* tests were used firstly. When variances were equal, a least significant difference (LSD) was used. α=0.05 was taken as the standard.

## Results

***Morphological characteristics and identification of BMSCs***

Primary passage of BMSCs had small nuclei and began to attach when cultured for 12 hr: part of the cell bodies spread and clung to the growth plane of the culture flasks. After 4 days, primary cells proliferated rapidly and protuberances with varying size and length were stretched out from the cell bodies. After 7 days, cells were mainly short shuttle-shaped, oval and irregularly-shaped ones were also visible, which were similar to fibroblasts in appearance. After passage, the cultured cells showed the shape of fusiform and arranged consistently.

**Figure 1.**
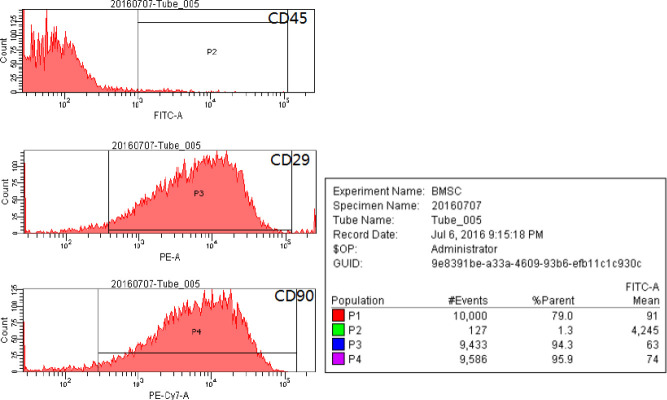
Flow cytometry results of bone marrow mesenchymal stem cells (BMSCs) labeled for cell-surface markers

FCM results indicated that the positive rates of CD29, CD45, and CD90 were 94.3%, 1.3%, and 95.9%, respectively. It revealed that these cells expressed stromal cell antigen rather than hematopoietic stem cell antigen. These results confirmed the characteristics of mesenchymal stem cells (Figure 1).

**Figure 2:**
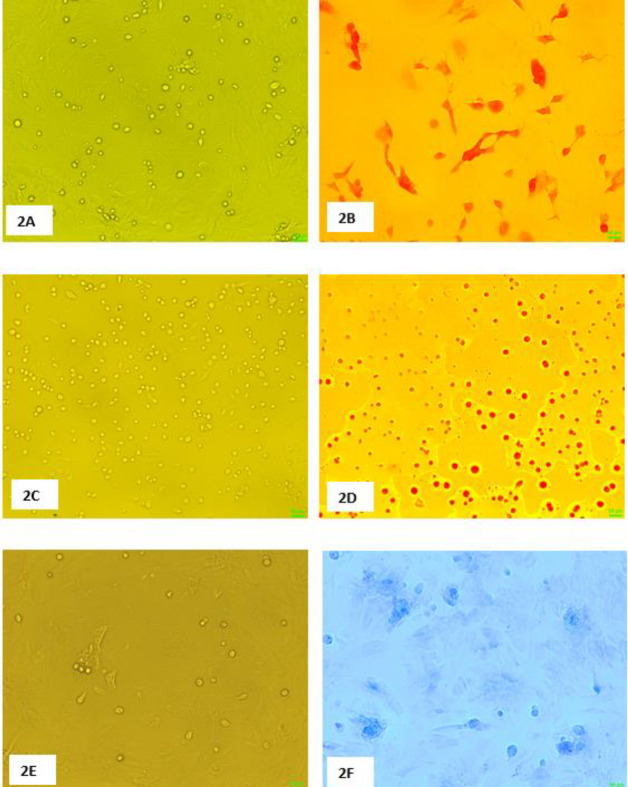
Identification of bone marrow mesenchymal stem cells (BMSCs) multidirectional differentiation ability

During osteogenic induction, BMSCs gradually transformed to polygonal or irregular-shaped cells and grew intensively (Figure 2A). The induced cells were positive for alizarin red staining after 21 days (Figure 2B). During adipogenic induction, small lipid droplets accumulated and became larger gradually in some cells (Figure 2C). The induced cells were positive for oil red O staining after 14 days (Figure 2D). During cartilage induction, the local part of BMSCs gradually changed into round or oval (Figure 2E). After 14 days, the cells and its related structures were positive for alcian blue staining (Figure 2F).

***Transduction and identification***

**Figure 3.**
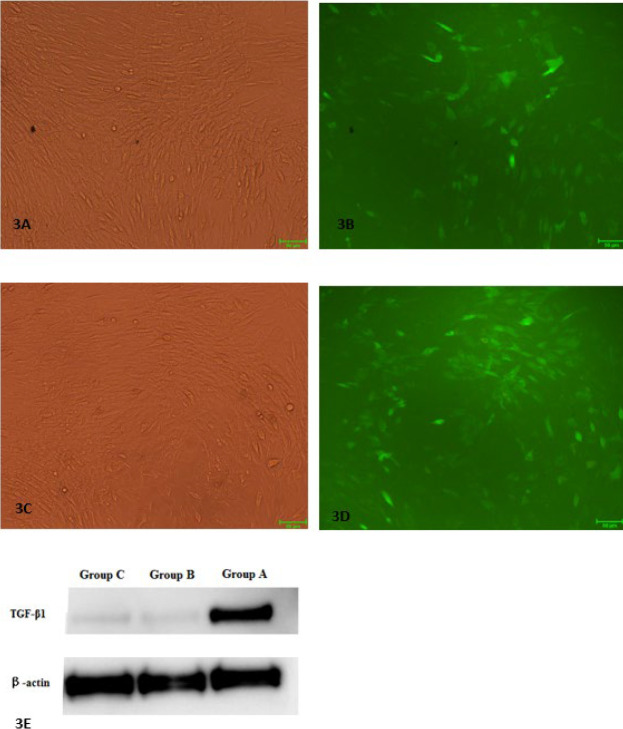
Morphologic changes of bone marrow mesenchymal stem cells (BMSCs) after transduction for 96 hr

The fluorescence intensity of cells in each group was the strongest at MOI of 150 (Figures 3A-3D). ELISA detection indicated that *in vitro *TGF-β1 secretion in BMSCs transducted with TGF-β1 was significantly higher than those in BMSCs transducted with empty lentivirus (when MOI=100: 1.315±0.128 ng/ml vs 0.875±0.082 ng/ml, *P*<0.05; when MOI=150: 2.055±0.188 ng/ml vs 1.395±0.134 ng/ml, *P*<0.05; and when MOI=200: 1.735 ±0.162 ng/ml vs 1.105±0.103 ng/ml, *P*<0.05), with no further secretion increase when MOI increased from 150 to 200 (*P*>0.05). 

Western blot results indicated that after 96 hr of transduction, cells in Group A (Lenti-TGF-β1-GFP) can express TGF-β1 while there was no expression of TGF-β1 in Group B (Lenti-empty vector-GFP) or Group C (BMSCs blank culture group) (Figure 3E).

***Analysis of BMSCs-cardiac differentiation after cells transducted with TGF-β1***

**Figure 4:**
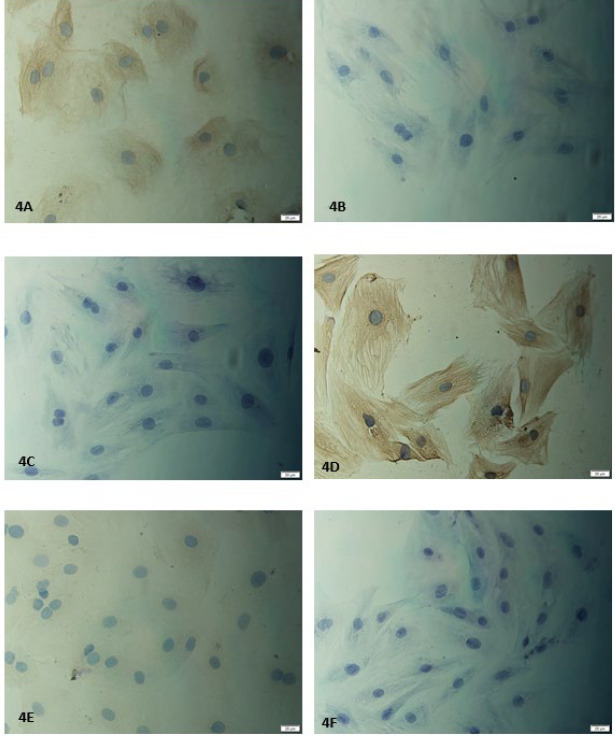
Immunohistochemical staining results of bone marrow mesenchymal stem cells (BMSCs) in each group

The results of immunocytochemistry showed that compared with Group B (Lenti-empty vector-GFP) or Group C (BMSCs blank culture group) (Figure 3E), BMSCs in Group A (Lenti-TGF-β1-GFP) could be identified by the positive expression of cTnI and Cx43 (*P*<0.05) (Figures 4A-4E).

**Figure 5:**
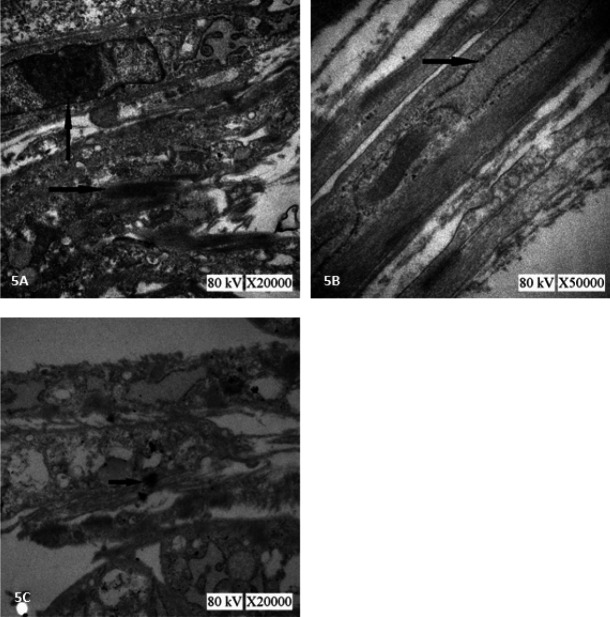
Morphologic changes of bone marrow mesenchymal stem cells (BMSCs) in group A (lenti-TGF-β1-GFP) by transmission electron microscopy (TEM)

TEM revealed organelles such as a mass of mitochondria and rough endoplasmic reticulum, meanwhile, gap junctions were also visible in cell cytoplasm of Group A (Lenti-TGF-β1-GFP). In addition, myofilament arranged parallel in the cytoplasm. There was usually a nucleus in the center of the cell. These ultrastructural characteristics of BMSCs in Group A (Lenti-TGF-β1-GFP) were similar to myocardial cells (Figure 5).

***Analysis of the relationship between myocardial differentiation of BMSC transducted with TGF-β1 and Wnt/β-catenin signaling pathway using Western blot***

**Figure 6:**
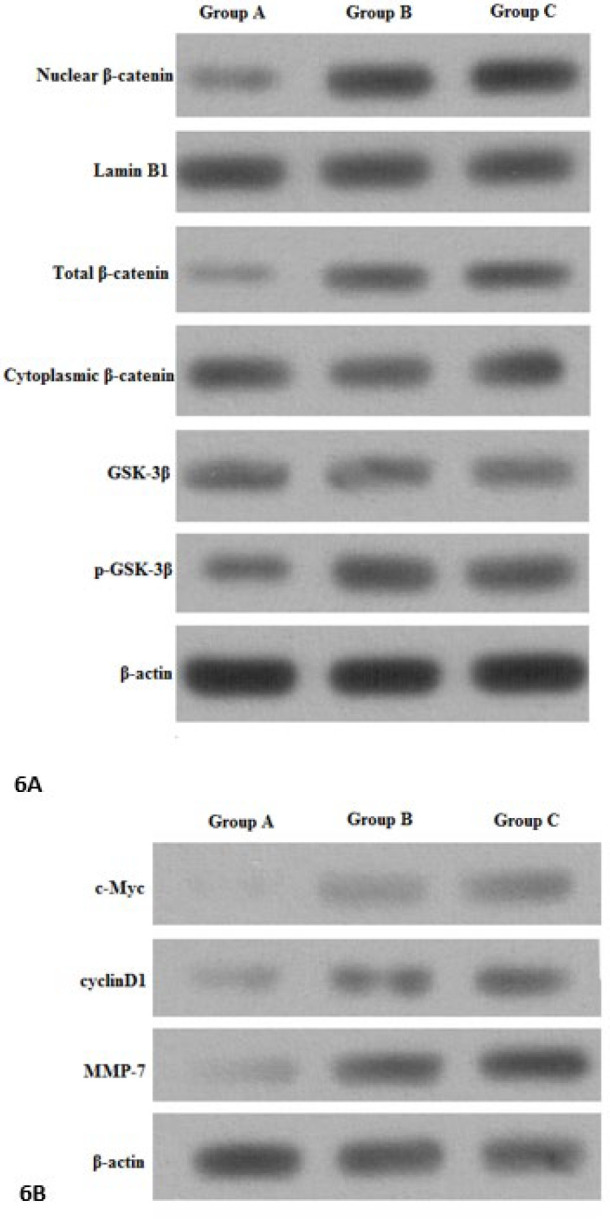
Western blot results of bone marrow mesenchymal stem cells (BMSCs) in each group

Following transduction of TGF-β1, the levels of intranuclear and total β-catenin were all significantly decreased in BMSCs (*P*<0.05). However, cytoplasmic β-catenin levels were largely unchanged. In addition, levels of GSK-3β were largely unchanged in BMSCs, whereas phosphorylated GSK-3β levels were significantly decreased in BMSCs (*P*<0.05, Figure 6A). MMP-7, c-Myc, and cyclinD1 protein expression in Group A (Lenti-TGF-β1-GFP) was all significantly lower than that in the blank culture group (Figure 6B).

***Analysis of the relationship between Wnt/β-catenin signaling pathway and myocardial differentiation of BMSC transducted with TGF-β1using RT-qPCR***

**Figure 7:**
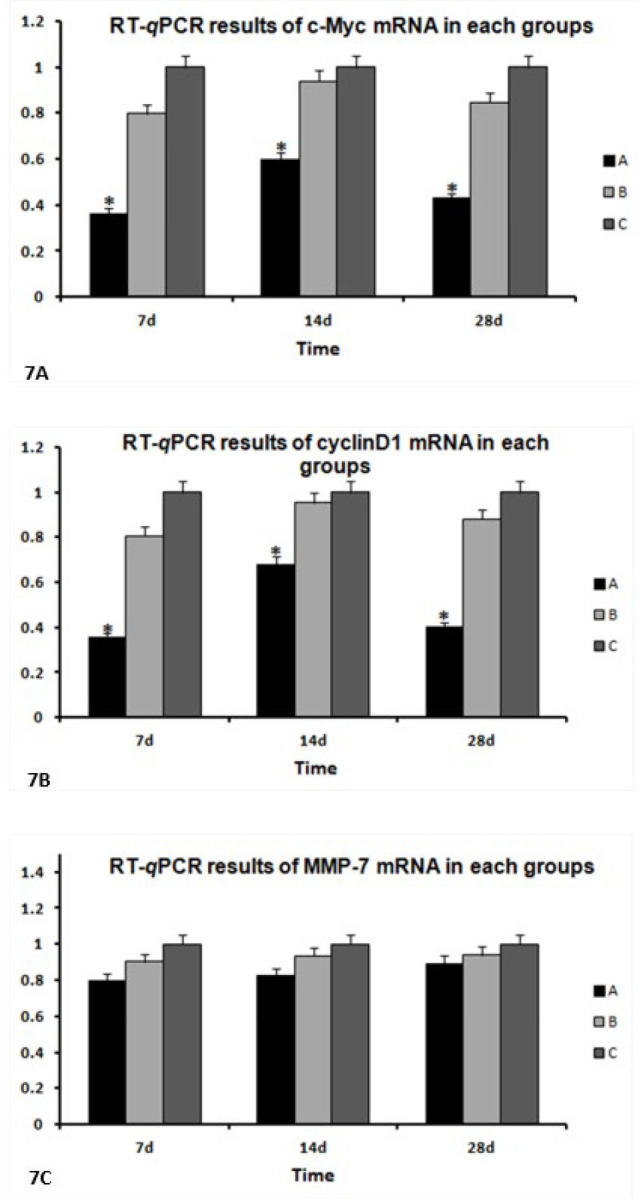
Real time fluorescence quantitative PCR (RT-qPCR) results of c-myc, cyclinD1 and MMP-7 mRNA

RT-*q*PCR results showed that at the 14th day after transduction, MMP-7, cyclinD1, and c-Myc mRNA expression was the strongest in each group. The expression of c-Myc and cyclinD1 mRNA in Group A (Lenti-TGF-β1-GFP) was lower than that in the blank culture group, respectively. At the 14th day after transduction, for cyclinD1 mRNA, the level in Group A (Lenti-TGF-β1-GFP) was 0.68-fold lower than that in the blank culture group. For c-Myc mRNA, it was 0.61-fold lower than that in the culture blank group (*P*<0.05, Figures 7A-7C).

***Analysis of the effect of LiCl on the expression of*** ***β*** ***-*** ***catenin*** ***and p-*** ***β*** ***-*** ***catenin in different*** ***groups using Western blot***

**Figure 8:**
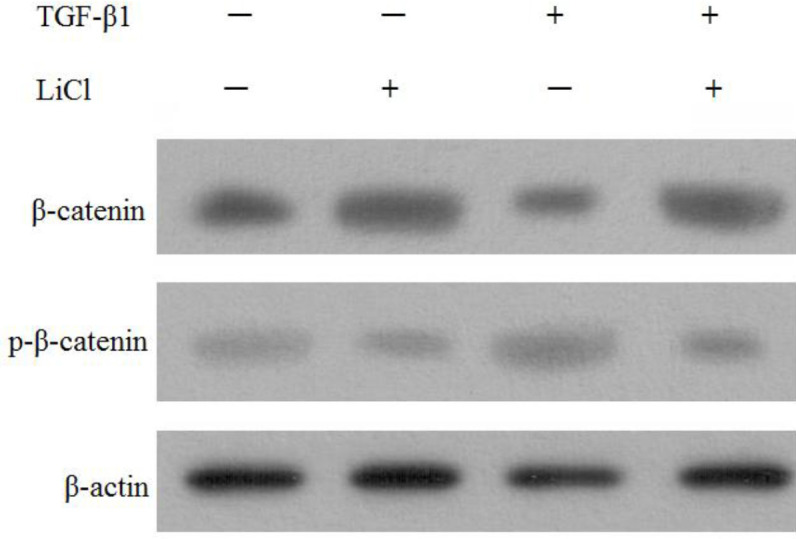
Western blot results of β-catenin and p-β-catenin protein of bone marrow mesenchymal stem cells (BMSCs) in each group

Compared with the blank culture group, β-catenin expression was decreased in single transduction group while phosphorylated β-catenin levels were increased (*P*<0.05). However, β-catenin expression in the LiCl group and transduction+LiCl group was all increased, while phosphorylated β-catenin levels were decreased (*P*<0.05, Figure 8).

**Table 1:** c-myc, cyclinD1, MMP-7, and GAPDH primers used for real time fluorescence quantitative PCR (RT-qPCR)

	primer
*c-* *m* *yc*	Forward: 5’- TTGAACGGACAGGATGTAGGC-3’
	Reverse: 5’- GAGGAGAAACGAGCTGAAGCG-3’
*cyclinD1*	Forward:5’-CCCTCCGTTTCTTACTTCA-3’
	Reverse: 5’-ACCTCCTCTTCGCACTTC-3’
*MMP-* *7*	Forward:5’-CCTTCACGACTCTAAAACAAA-3’
	Reverse:5’-ACATCTGGCACTCCACAC-3’
*GAPDH*	Forward: 5’-TGACTCTACCCACGGCAAGTTCAA-3’
	Reverse: 5’-ACGACATACTCAGCACCAGCATCA-3’

A, morphologic change of BMSCs which were induced under osteogenic differentiation conditions; B, the induced BMSCs were detected by Alizarin red staining; C, morphologic change of BMSCs which were induced under adipogenic differentiation conditions; D, the induced BMSCs were detected by Oil Red O staining; E, morphologic change of BMSCs which were induced under chondrogenic differentiation conditions; F, the induced BMSCs were detected by Alcian blue staining; magnification: ×100

A, BMSCs in group A by inverted microscope; B, fluorescent expression of BMSCs in group A; C, BMSCs in group B by inverted microscope; D, fluorescent expression of BMSCs in group B; E, detection of transforming growth factor beta 1(TGF-β1) protein expression by Western blot; magnification: ×100; group A: lenti-TGF-β1-GFP; group B: lenti-empty vector-GFP; group C: BMSCs blank culture group; MOI =150

A, cTnI expression in group A; B, cTnI expression in group B; C, cTnI expression in group C; D, cx43 expression in group A; E, cx43 expression in group B; F, cx43 expression in group C; magnification: ×400; group A: lenti-TGF-β1-GFP; group B: lenti-empty vector-GFP; group C: BMSCs blank culture group

A, myofilaments (→) were found in parallel in the cytoplasm. (↑) is the nucleus. magnification: ×20,000; B, rough endoplasmic reticulum (→) in the cytoplasm; magnification: ×50,000; C, gap junction (→) between cells; magnification: ×20,000

A, β-catenin, GSK 3β, and p- GSK3β protein expression; B, c-myc, cyclinD1, and MMP-7 protein expression; group A: lenti-TGF-β1-GFP; group B: lenti-empty vector-GFP; group C: BMSCs blank culture group

A, c-myc mRNA; B, cyclinD1 mRNA; C, MMP-7 mRNA; group A: lenti-TGF-β1-GFP; group B: lenti-empty vector-GFP; group C: BMSCs blank culture group; *P<0.05 vs BMSCs blank culture group

Compared with the BMSCs blank culture group, β-catenin expression was decreased significantly in single transfection group while phosphorylated β-catenin expression was increased. β-catenin expression in LiCl group and transfection+LiCl group was increased, while phosphorylated β-catenin expression was decreased

## Discussion

The main findings from the study are as follows: (i) TGF-β1 increases differentiation of BMSCs towards CMCs; (ii) in the process of enhancing differentiation of BMSCs towards CMCs by transducting with TGF-β1 gene, it may be through promoting the phosphorylation of β-catenin; (iii) it may also be through inhibiting c-Myc, cyclinD1, and MMP-7 expression of Wnt/β-catenin signaling.

Regeneration of the myocardium in which irreversible cell loss occurs as a result of MI and other ischemic heart diseases has been an ideal goal of cardiologists (25). As for the abilities of self-renewal and multiple differentiations potential, BMSCs have been demonstrated to differentiate towards CMCs (6, 7). TGF-β1 is an efficient regulator of the extracellular matrix. It can also improve BMSCs cardiogenic differentiation (26). TGF-β1 shows a biphasic role in many physiological and pathological processes, for instance, TGF-β1 with low concentrations significantly increase, whereas with high concentrations decrease endothelial cells vascular invasion (27). The results of our earlier papers (23), confirmed that TGF-β1 treatment can improve BMSCs cardiogenic differentiation, and the optimal concentration was 5 ng/mL. This process may be achieved by autocrine or paracrine modes. 

Lentiviral vectors can be used to stably transduct cells and exhibit long-term expression (28). Accordingly, we first constructed TGF-β1 co-transducted rat BMSCs to detect the effects of TGF-β1 overexpression on cardiogenic differentiation of BMSCs. The results displayed that after 96 hr of transduction, cells in Group A (Lenti-TGF-β1-GFP) can express TGF-β1 while there was no expression of TGF-β1 in Group B (Lenti-empty vector-GFP) or Group C (BMSCs blank culture group). Furthermore, the results of immunocytochemistry showed that compared with Group B (Lenti-empty vector-GFP) or Group C (blank culture group of BMSCs), BMSCs in Group A (Lenti-TGF-β1-GFP) could be identified by positive expression for cTnI and Cx43. As a diagnostic biomarker in acute coronary syndromes patients, cTnI is exclusively present in the cardiac muscle. Cell gap junction is also an important cardiomyocyte feature (29) and Cx43 is a vital component of cell gap junction. TEM results also revealed that the ultrastructural characteristics of BMSCs in Group A (Lenti-TGF-β1-GFP) were similar to myocardial cells. All results displayed that TGF-β1 is a factor of BMSCs differentiated toward CMCs. The results were consistent with those of another study (30). 

With the rapid development of cell basic research, cell-based therapy has been used in a variety of clinical fields such as in orthopedics, central nervous system, and cardiovascular system, and presents a promising future prospect (31). BMSCs cardiogenic differentiation is a complex process involving several different signals. Further knowledge of biochemical pathways including molecule signaling pathways can provide more insights into differentiation mechanism of BMSCs. 

As is well known, the canonical Wnt/β-catenin pathway can regulate the equilibrium between cell differentiation and proliferation. For β-catenin, its stabilization and nuclear translocation can lead to Wnt target genes transactivation (32). When Wnt ligand is absent, β-catenin is recruited into a ‘destruction complex’ (including Axin, APC, CK1, and GSK-3β), where it is phosphorylated, degraded to keep it at low levels (33, 34). When Wnt presents, the complex is inhibited. β-Catenin translocates to the nucleus where it up-regulates a wide range of target genes (c-myc, cyclin D1, MMP-7, etc.).

β-catenin signaling is important in cardiac remodeling (35-37). However, an opposite role has been observed for β-catenin in the cardiac response to angiotensin II infusion (38). These contradictory findings uncover the complexity of the Wnt pathway in terms of its role in different disease stages as well as cell-autonomous vs non-cell-autonomous actions. The other limitation is that most investigations have only explored the short-term effects of β-catenin activation and inhibition in the heart. Therefore, in the heart, low level and short-term activation of β-catenin may not be harmful; its prolonged and sustained stimulation is potentially detrimental and can lead to cardiac dysfunction. Long-term follow-up investigations in animal models, as well as human clinical trials, are required to determine whether Wnt or β-catenin inhibition can prevent adverse cardiac remodeling and pump dysfunction in a variety of heart diseases (39). GSK-3β is inhibited by phosphorylation at specific serine residues (40) and in the ‘destruction complex’, Axin interacts with GSK-3β, which facilitates β-catenin phosphorylation (41). Then phosphorylated β-catenin is ubiquitinated, which leads to its proteasomal degradation rapidly. In this study, following transduction of TGF-β1, intranuclear and total β-catenin levels were all decreased in BMSCs. However, cytoplasmic β-catenin levels were largely unchanged. As predicted, the level of GSK-3β was largely unchanged in BMSCs, whereas phosphorylated GSK-3β was significantly decreased in BMSCs. When given LiCl, one of the activators of Wnt/β-catenin pathway to BMSCs transducted with TGF-β1, β-catenin was increased, while phosphorylated β-catenin was decreased. Our results demonstrate that in the process of enhancing differentiation of BMSCs towards CMCs by transducting with the TGF-β1 gene, it may be through promoting the phosphorylation of β-catenin.

As a target of β-catenin activated transcription (39), c-Myc also induces the expression of cyclinD1, which is another target of β-catenin induced transcription (42). cyclinD1 is a regulatory factor for cyclin dependent kinases. MMP-7 can degrade the extracellular matrix (43). In this study, MMP-7, cyclinD1, and c-Myc protein expression in BMSCs transducted with TGF-β1 was lower than that in the blank culture group. Therefore, it revealed that in the process of enhancing differentiation of BMSCs towards CMCs by transducting with the TGF-β1 gene, it may also be through inhibiting c-Myc, cyclinD1, and MMP-7 expression of Wnt/β-catenin signaling.

## Conclusion

This study indicated that TGF-β1 promotes BMSCs cardiomyogenic differentiation by promoting the phosphorylation of β-catenin and inhibiting cyclinD1, MMP-7, and c-Myc expression in the Wnt/β-catenin signaling pathway.
